# Periorbital necrotizing fasciitis: the Manchester experience

**DOI:** 10.1186/s12886-025-04062-3

**Published:** 2025-04-17

**Authors:** Abigail Hopkins, Jonathan K. Y. Ng, Jae Yee Ku, Adam Bull, Qistina Pilson, Anne Cook

**Affiliations:** 1 Manchester Royal Eye Hospital, Oxford Road, Manchester, M13 9WL UK; 2 Department of Eye and Vision Science, Institute of Life Course and Medical Sciences, Faculty of Health & Life Sciences, University of Liverpool, William Henry Duncan Building, 6 West Derby Street, Liverpool, L7 8TX UK; 3 Stepping Hill Hospital, Hazel Grove, Poplar Grove, Stockport, SK2 7JE UK

**Keywords:** Periorbital, Necrotizing, Fasciitis, Infection, Soft tissues

## Abstract

**Background:**

To describe the presentation and management of patients with periorbital necrotizing fasciitis (PONF) through an observational retrospective case series. The clinical notes of twelve consecutive patients managed by the Oculoplastic and Orbital Service and Maxillofacial Service of the Manchester University NHS Foundation Trust between 2018 and 2023 were reviewed. Five of these patients were contactable and gave informed consent for inclusion in the study.

**Methods:**

Retrospective review of patient characteristics, risk factors, clinical findings, Laboratory Risk Indicator for Necrotizing Fasciitis (LRINEC) score, imaging results, microbiology and histology results, patient management and mortality.

**Results:**

The majority of the patients were male (*n* = 3) with a median (IQR) age of 63 (51–71) years. The median (IQR) number of risk factors per patient was 1 (0–1.5). All patients had periorbital swelling at presentation. Median (IQR) LRINEC score was 5 (3–8.5). Group A Streptococcus was isolated from at least one sample (wound swab, tissue sampling, blood culture) in 4 cases. Histology was consistent with PONF in the remaining case. All patients received intravenous antibiotics and had between 1 and 4 surgical debridements. The median (IQR) time from the onset of symptoms to antibiotic treatment was 24 (17–42) hours, and the time of suspected diagnosis to debridement was 4 (2.3–6) hours. The median (IQR) final best recorded visual acuity (BRVA) was 2.0 (0.23–3) logMAR. Three patients developed orbital compartment syndrome; 2 of these had a final BRVA of no perception of light (NPL). The median (IQR) time from the initial surgery to the most recent follow-up was 4 (2.5–42) months. There was no mortality.

**Conclusions:**

This study showed no mortality in PONF due to early antibiotic treatment and surgical debridement. A high index of clinical suspicion for PONF using the LRINEC score and other parameters combined with a low threshold for treatment should be maintained, especially in high-risk groups. Urgent referral to specialist surgical teams to ensure prompt diagnosis and treatment is essential to optimise outcomes in the face of this destructive infection.

## Background

Necrotizing fasciitis (NF) is a rare and severe infection characterised by rapidly spreading necrosis of the soft tissues. Periorbital necrotizing fasciitis (PONF) is a very rare condition with a United Kingdom (UK) incidence of 0.24 cases per million per year [1]. The mortality rate from NF is high at 32.3% [2], whilst PONF has a lower mortality rate than elsewhere in the body with rates ranging from 3% to 14.4% [3–7]. PONF also carries immense morbidity in terms of visual loss, facial deformity, functional and psychological issues long term [7, 8].

Early diagnosis and treatment, including intravenous (IV) antibiotics and surgical debridement, are key in reducing morbidity and mortality [4]. However, early signs including swelling, erythema and pain may be similar to other less aggressive soft tissue infections such as preseptal cellulitis. Although rapid progression to extensive necrosis of soft tissues and sepsis is likely to follow, the ambiguous early signs may lead to a delay in diagnosis and poor outcomes. Given this clinical dilemma, Wong et al. developed a novel diagnostic scoring system known as the Laboratory Risk Indicator for Necrotizing Fasciitis (LRINEC) score in 2004 for distinguishing NF from other soft tissue infections. The LRINEC score is based on laboratory tests routinely performed for the evaluation of severe soft tissue infections [9]. However, its utility as a predictor in PONF has been debated [10].

We present a series of 5 patients with PONF managed by a single, tertiary centre in Manchester, UK, over 5 years. We assessed the predisposing risk factors, presenting signs, laboratory findings, management and outcomes.

## Methods

Retrospective, observational case series of 5 consecutive patients with histologically confirmed PONF presenting to the Oculoplastics and Orbital Service and Maxillofacial Service of the Manchester University NHS Foundation Trust, UK, over the 5 years (2018–2023). Data collected included age, gender, date and time of presentation, risk factors (trauma, infection, alcoholism, immunosuppression, recreational drug abuse, concurrent infection, significant medical comorbidities), presenting signs, laboratory findings relevant for the LRINEC score (C reactive protein (CRP), white cell count (WCC), Haemoglobin (Hb), glucose, sodium, creatinine), imaging, management (including antimicrobial and surgical treatment), microbiology and histopathology results. Best recorded visual acuity (BCVA) was measured and reported as logMAR using 2 decimal points, with numeric substitutions of 2.0 and 3.0 for perception of light (PL) and no perception of light (NPL), respectively. All data analyses were performed using Excel (2016) and GraphPad Prism (version 9). Utilising the NHS Health Research Authority decision tool, our study was deemed not to require ethical approval due to its retrospective and observational nature. Informed consent was obtained from all patients.

## Results

### Baseline characteristics

Twelve patients with PONF over the specified period were identified, 7 were excluded as they were uncontactable, and therefore we were unable to obtain informed consent for inclusion. 5 patients were included. Most patients were male (3/5; 60%), and they had a median (IQR) age of 63 (51–71; range 42–75) years at presentation (Table 1).

**Table 1:** Patients’ demographics, risk factors, wound, blood culture and tissue biopsy results, Laboratory Risk Indicator for Necrotizing Fasciitis (LRINEC) score and antibiotics therapy

Patient	Age	Gender	Risk Factors	Wound Swab Results	Blood Culture results	Tissue Biopsy Results	Antibiotics/anti-viral therapy at presentation
1	59	M	PR3 ANCA positive vasculitis, Type 2 DM	NG	Gram-positive cocci. NG	NF^a^	Co-amoxiclav, metronidazole
2	63	F	Trauma	GAS	NG	NF^a^, GAS	Ceftriaxone, clindamycin
3	42	M	Nil	GAS	NG	NF^a^, GAS	Ceftriaxone, clindamycin
4	75	F	Right Herpes Zoster Ophthalmicus	GAS	NG	NF^a^, GAS	Ceftriaxone, clindamycin, gentamicin, aciclovir
5	66	M	Nil	SA	NA	NF^a^, GAS, SA	Ceftriaxone, clindamycin, metronidazole

*DM* Diabetes Mellitus, *NG* No growth, *NA* Not available, *GAS* Group A Streptococcus, *SA* Staphylococcus aureus

^a^Morphological appearances on histology consistent with necrotising fasciitis (NF)

### Risk factors

The median (IQR) number of risk factors per patient was 1 (0–1.5; range 0–2) (Table 1). Trauma was identified as a risk factor in one case. One patient was immunosuppressed on oral prednisolone for PR3-ANCA positive vasculitis, and one patient had suspected preceding right-sided Herpes Zoster Ophthalmicus (HZO) infection. Two patients were found to have no risk factors for PONF.

### Clinical findings at presentation

All patients had unilateral periorbital swelling at presentation (4 right side, 1 left side). Three patients had significant sloughing of periocular tissue and skin breakdown. Two patients (patients 3 and 5) presented with orbital compartment syndrome with raised intraocular pressures (IOP) and a relative afferent pupillary defect (RAPD). Patient 3 presented with BRVA of NPL, while patient 5 presented with BRVA of PL. BRVA at presentation was measured in 60% (3/5) of the cases. In cases where vision was not documented, this was due to the clinician’s inability to open the eye (despite the assistance of Desmarres retractors) due to substantial periocular swelling. Where BRVA at presentation was obtainable, it ranged from 1.0 logMAR to NPL.

### Laboratory findings/LRINEC

Data contributing to the LRINEC score were available in all patients. At presentation, median (IQR) CRP was 220.5 (47.1–340.3; range 10.5–359) mg/L, WCC 18.2 (12.3–31.5; range 11.4–34.9 × 10/uL), Hb 138 (121–147.5; range 116–150) g/l, glucose 7.6 (6.4–7.8; range 6.4–7.8) mmol/L, sodium 137.5 (132.5–138.8; range 131–139) mmol/L and creatinine 76.5 (56.8–103; range 54–108) mg/dl. Median (IQR) LRINEC score was 5 (2–7.3; range 1–8), and 2 patients had a score of 7 or greater.

### Imaging results

Computerised tomography (CT) imaging of the head and orbits was undertaken in all patients at presentation. Results showed soft tissue swelling in the periorbital region in all cases. The soft tissue inflammatory changes appeared to be pre-septal in 3 cases and post-septal in 2 cases. Intra-orbital gas was not noted in any of the cases included in this study.

**Fig. 1:**
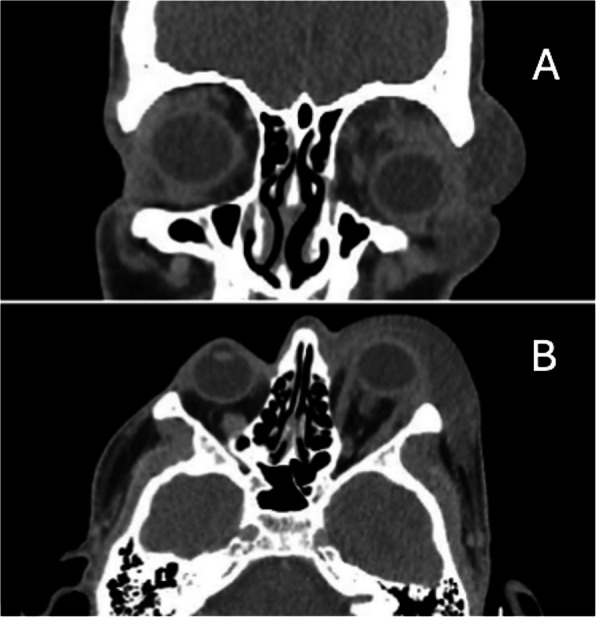
CT scan of patient 3 at presentation showing soft tissue thickening in the superior and lateral aspect of the left orbit, displacing the orbital structures inferiorly, shown in the coronal (**A**) and axial (**B**) sections

On CT imaging, patient 3 was found to have soft tissue thickening in the superior and lateral aspect of the orbit, displacing the orbital structures inferiorly (Fig. 1). It was deemed that these findings were likely to represent infection. He had no well-defined intraorbital fluid collection and no underlying sinusitis.

### Microbiology and histology results

Wound swabs and tissue sampling were undertaken in all patients (Table 1). Peripheral blood cultures were taken in 4 patients. Group A Streptococcus was isolated from wound swabs in 3 cases, tissue samples in 4 cases and no blood cultures. Group A Streptococcus was isolated from at least 1 sample in 4 cases. In the remaining case (Patient 1), although Gram, Grocott and PAS stains were negative for microorganisms, the overall morphological appearances on histopathology were consistent with NF.

### Antimicrobial treatment

At presentation, all patients received IV antibiotics. The choice of initial antimicrobial therapy at presentation was variable (Table 1). The time from patient-reported onset of symptoms to treatment with antibiotics was available in all cases (median 24, IQR 17–42, range 10–48 h) (Table 2). Two patients received a combination of IV ceftriaxone and clindamycin only. The remaining 3 patients received various combinations of IV antibiotics including co-amoxiclav (*n* = 1), metronidazole (*n* = 1), clindamycin (*n* = 2), gentamicin (*n* = 1) and ceftriaxone (*n* = 2). One patient was also commenced on IV aciclovir due to a suspected herpes zoster infection.

**Table 2:** Patients’ best recorded visual acuity (BRVA), time to treatments, number of debridements, complications, reconstructive procedures and follow-up duration

Patient	Laterality	BRVA at presentation (logMAR)	Final BRVA (logMAR)	Onset of symptoms to antibiotic therapy (hours)	Clinical diagnosis PONF to surgery (hours)	Number of debridement(s)	Complications	Initial surgery to last follow-up (months)	Reconstructive procedures
1	Right	UO	0.1	10.0	2.5	3	Lagophthalmos, Ectropion	32	Right upper and lower lid integra graft, right upper lid FTSG
2	Right	1.0	NPL	24.0	4.0	4	OCS^a^, Lagophthalmos, EK	51	Temporalis flap, right upper lid SSG, right upper lid revision with orbicularis flap and skin graft, right upper lid skin/orbicularis transposition flap
3	Left	NPL	NPL	23.0	2.0	4	OCS^b^, Lagophthalmos, EK	4	Left upper lid integra graft followed by FTSG
4	Right	UO	0.36	48.0	8.0	1	Lagophthalmos, EK	1	Right lateral orbital orbicularis propeller flap
5	Right	PL	PL	36.0	4.0	2	OCS^b^	0	Right upper and lower lid integra graft, right upper and lower lid SSG

*PONF* Periorbital necrotizing fasciitis, *UO* Unable to open, *PL* Perception of Light, *NPL* No perception of light, *OCS* Orbital compartment syndrome, *EK* Exposure keratopathy, *SSG* Split thickness skin graft, *FTSG* Full thickness skin graft

^a^Post operative OCS

^b^OCS at presentation

### Surgical management

All patients underwent surgical debridement with a multi-disciplinary team approach. The maxillofacial team was involved in 4 cases, and the Ear, Nose and Throat (ENT) team was involved in 2 cases. The time from clinical diagnosis of PONF to the first debridement was recorded in all 5 cases with a median (IQR) of 4 (2.3–6; range 2–8) hours. Patients underwent between 1 and 4 surgical debridements (median 3, IQR 1.5–4). In all cases, necrotic tissue was described intraoperatively, and debridement down to healthy bleeding tissue was achieved.

**Fig. 2:**
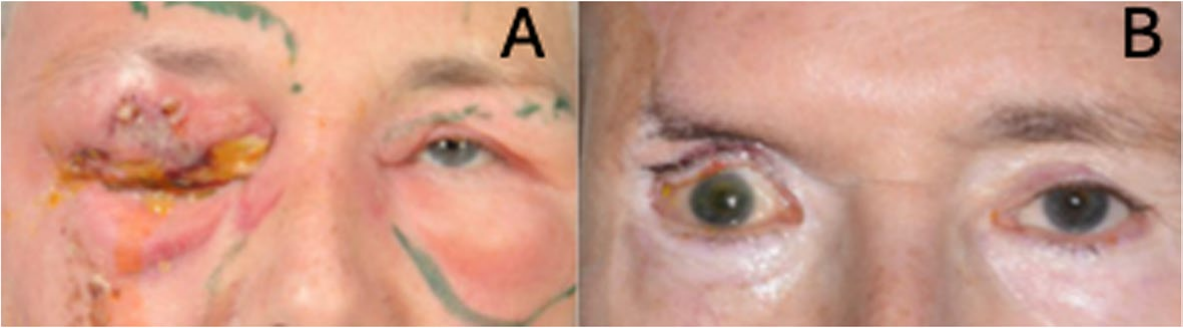
Patient 2 at presentation showed right-sided dusky peri-orbital erythema, necrosis and sloughing of right upper eyelid skin (**A**), and at their most recent follow-up, following debridement and reconstruction, where there is apparent right upper lid tissue loss (**B**)

**Fig. 3:**
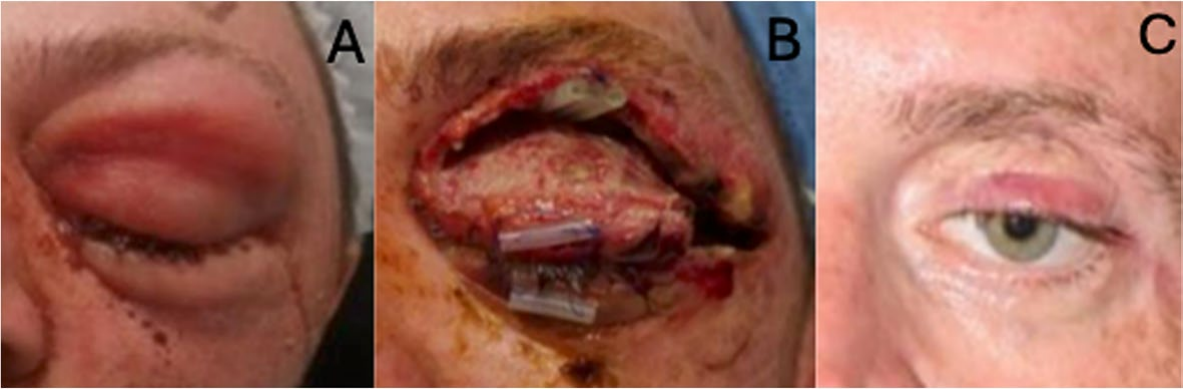
Patient 3 prior to their first debridement showed significant left-sided peri-orbital swelling (**A**), before their third exploration and debridement showing the extent of debrided tissues with tarsorrhaphy and drain in situ (**B**) and at their most recent follow-up following debridement and reconstruction showing slight thickening of the upper lid graft and hypoglobus (**C**)

**Fig. 4:**
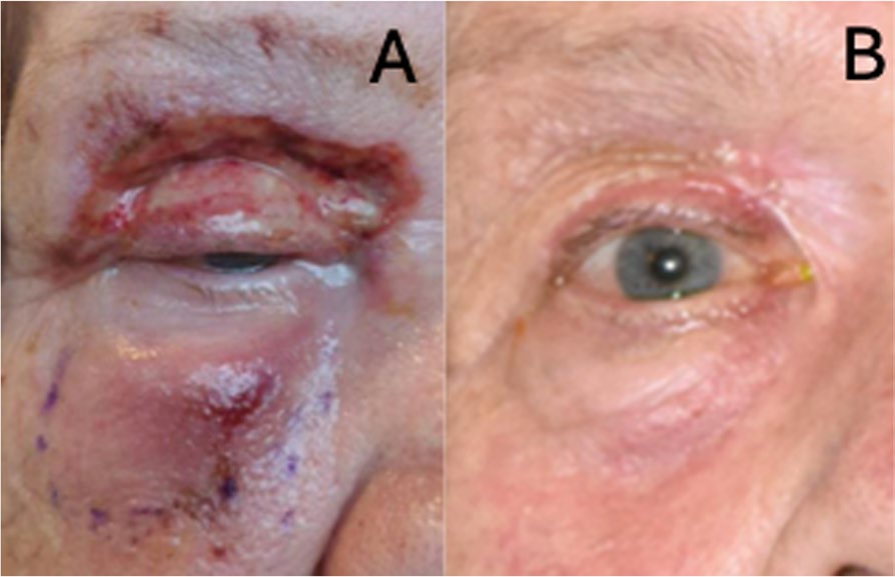
Patient 4 at presentation showed right-sided slightly dusky peri-orbital erythema with sloughing of upper lid tissue (**A**). At their most recent follow-up, following debridement and reconstruction, showed slight thickening and contracture of the flap with medial webbing (**B**)

All patients required further reconstructive procedures (Table 2). Skin grafting was involved in reconstruction in 4 cases. Pre and post-operative images from patients 2, 3 and 4 are shown in Figs. 2, 3 and 4, respectively.

### Post-operative / follow-up

The median (IQR) time from initial surgery to their most recent follow-up was 4 (2.5–42; range 1–51) months (Table 2). The median (IQR) final BRVA (BRVA at their most recent follow-up) was 2 (IQR 0.23–3, range 0.10–3.0) logMAR. Patient 2 developed post-op orbital compartment syndrome following their first debridement. This patient underwent urgent canthotomy and cantholysis, followed by urgent orbital exploration and medial wall decompression along with further debridement. In this case, the final BRVA did not improve from NPL, and there was significant upper lid tissue loss and lagophthalmos (Fig. 2).

Most patients (80.0%; 4/5) had some degree of lagophthalmos at their most recent follow-up, and 60% (3/5) had evidence of exposure keratopathy. No patients required evisceration or enucleation. The mortality rate amongst the studied cohort was 0%.

## Discussion

Risk factors for the development of PONF include, but are not limited to, trauma, recent surgery, co-existing infection, alcoholism, drug abuse, diabetes, rheumatological disease, chemotherapy, immunosuppression, malignancy, renal failure and systemic malignancy [7]. In our series, most patients had at least one risk factor for the development of the disease.

It is well recognised that drug or alcohol misuse predisposes patients to the development of necrotising fasciitis. Unfortunately, due to issues tracing patients from this cohort to obtain informed consent, 5 patients with a history of drug or alcohol misuse had to be excluded from the study. A study by Yii et al. examined the relationship between alcohol intake and infection with NF. The study found that the incidence of NF in alcohol misuse disorder was nearly 7.73 times higher than in controls [11]. A number of theories exist to explain this correlation. First, chronic, heavy consumption of alcohol affects numerous components of the immune system, ultimately causing immunosuppression, leading to increased susceptibility to all types of infection [12]. One study suggested that increased alcohol exposure is associated with poor wound healing and increased susceptibility to Group A Streptococcal infection [13]. In addition, studies have shown that intoxication with alcohol significantly increases the risk of trauma and hence soft tissue injury, therefore increasing the risk of development of secondary infection [14, 15]. Alcoholism is known to be associated with early self-discharge and poor compliance with treatment advice [11, 16, 17]. Delays in receiving appropriate treatment for PONF are likely to lead to more rapid progression, increased severity of disease, more intensive management and higher rates of morbidity and mortality. This association between drug and alcohol misuse and PONF is not accurately portrayed in this study due to patient tracing issues.

The majority of patients in our cohort presented with a short history of unilateral periocular swelling. Periocular swelling is a common, non-specific symptom which may be associated with many less serious infective conditions, such as pre-septal cellulitis. Given the severity of the swelling in our cohort, the differential diagnosis in most patients was orbital cellulitis, and urgent Ophthalmology input was sought. Early commencement of antimicrobial therapy for probable orbital cellulitis was common and preceded the clinical diagnosis of PONF in all cases. In most cases, clinical diagnosis of PONF was delayed until the patient was referred and transferred from the presenting trust to our centre for ophthalmic assessment, which caused a delay in diagnosis and appropriate management for PONF.

Patient 4 of this case series (Table 1) presented to a different centre with unilateral periocular swelling and scab-like lesions in the periocular region (Fig. 4). She was initially treated for HZO for around 12 h, followed by a rapid increase in periocular swelling and systemic deterioration with the development of sepsis. Several cases of NF following Herpes Zoster infection have been reported [18–23]. Unfortunately, at the centre to which she presented, patient 4 had no microbiological testing at the onset of the initial vesicular rash, and no viral Polymerase Chain Reaction (PCR) was sent as the disease progressed. It remains unclear if the presumed preceding viral infection led to the development of PONF or if there was a misdiagnosis at presentation. This patient was not known to be immunosuppressed. She only required 1 surgical debridement and 1 reconstructive procedure.

Given the issues mentioned previously regarding the ambiguous signs and symptoms of PONF, the LRINEC score developed by Wong et al. to distinguish NF from other soft tissue infections has been applied to diagnose PONF [9]. However, the LRINEC score was developed based on a retrospective observational study, and it was not designed specifically for diagnosing PONF. Tambe et al. found that in their case series of 11 patients with PONF, the LRINEC score did not correlate with disease severity [10].

In this case series, we found that there was incomplete LRINEC scoring data, with a missing glucose value in 1 of our cases. Patient 3 had a low score of 1 despite a confirmed diagnosis of PONF. Since the LRINEC score is considered unreliable in this setting [10], the calculation of the LRINEC score at presentation should be used only as an aid to the diagnosis of suspected PONF and interpreted with caution. The score must be coupled with a careful review of suggestive clinical signs and symptoms, and any suspicion of PONF should still trigger an urgent Ophthalmology review.

Previous studies have also advocated the use of CT scanning to support the diagnosis of PONF [4, 7]. Signs of NF on CT scanning include fascial thickening, fat stranding, gas tracking along fascial planes and abscesses [4, 24–26]. With none of our cases demonstrating gas on their initial CT scan, our results suggest that gas on the CT scan may not be a sensitive marker of PONF. CT scanning, however, is still useful in cases of suspected severe periocular infection to assess the extent of the infection and assist in surgical planning [27].

It is well-recognised that PONF has a more favourable mortality rate than the same infection elsewhere in the body [28]. It has been postulated that the reason for the superior prognosis in PONF is the earlier presentation, given that the periocular region is highly visible, and swelling may lead to a reduction in vision. Secondly, antibiotics may have improved penetration to the periocular area, given the extensive blood supply of this region. This may result in more rapid uptake by tissues surrounding the necrotic areas, leading to slower dissemination of the infection. Finally, the orbital septal integrity may prevent or delay posterior orbital spread following pre-septal infection [3]. In our study, we found a mortality rate of 0%, which was lower than that of some similar UK studies [1, 3]. This observation may be related to the fact that a number of patients with multiple risk factors, including trauma and drug or alcohol misuse, were excluded from the study as they were uncontactable to provide consent, leaving a potentially healthier cohort with fewer comorbidities.

The final visual outcome in our cohort was variable. Two patients had a final BRVA of 0.36 logMAR or better, while 3 eyes had final visual acuity of PL or NPL secondary to orbital compartment syndrome. The development of orbital compartment syndrome secondary to PONF is not well documented in the literature. Given our findings, it is pertinent that patients should be closely monitored for this specific complication during the disease course. Pronounced periocular swelling with a tense orbit, indicative of orbital compartment syndrome, is an ophthalmic emergency and requires urgent diagnosis and management with a canthotomy and cantholysis to preserve sight. Ophthalmologists and other front-line health care professionals should recognise this complication and perform a canthotomy and cantholysis urgently to preserve sight.

All our patients received IV antibiotics and underwent surgical debridement. Many patients had multiple debridements (range 1–4). While it is not possible to determine if antibiotics or surgical debridement alone leads to a better outcome, most studies use a combination of both [3, 7, 10]. In this series, we noted much variability in antimicrobial therapy commenced at presentation. There are a number of reasons for this lack of consistency. In most cases, antibiotics were commenced at peripheral units prior to the patient being transferred to our tertiary centre for ongoing management. At these units, there are likely to be different guidelines in place for antimicrobial treatment. In addition, diagnostic uncertainty is likely to lead to inconsistency in antimicrobial prescription. As we have previously discussed, PONF is often misdiagnosed as orbital cellulitis. In many cases, initial antimicrobial treatment was presumably targeted towards this diagnosis. Our local microbiology guideline advises IV meropenem, clindamycin and gentamicin and discussion with a consultant microbiologist for suspected cases of PONF. On transfer to our centre, all cases were discussed with our local microbiology team for expert advice and antibiotic treatment was appropriately modified on an individualised basis depending upon current and previous antibiotic usage, patient allergies and available microbiology results. In cases of suspected PONF, appropriate broad-spectrum antibiotic therapy is paramount [7]. The variability in antimicrobial prophylaxis in our series highlights the need for a robust antimicrobial guideline which all clinicians are aware of and can refer to for suspected PONF cases.

Given the necrotic nature of NF, a combination of antimicrobial therapy and early debridement, instead of antimicrobial therapy alone, is likely to improve patient outcomes as antibiotic penetration into necrotic tissues may be limited. Therefore, previous studies have historically advocated early and extensive debridement down to healthy tissue in cases of PONF [4, 7]. More recent studies suggest that antibiotic therapy alone may offer comparable results in terms of morbidity and mortality [3, 29, 30]. However, the evidence here remains limited, and this approach should be taken with much caution.

We are aware of reports of adjunctive use of IV immunoglobulin in PONF [31]. However, as evidence for its efficacy is lacking, IV immunoglobulin was not used as a treatment modality in our cohort. Adjunctive steroid was also not utilised in our patient cohort.

## Conclusion

In conclusion, this study showed no mortality in PONF due to early antibiotic treatment and surgical debridement. A high index of clinical suspicion for PONF using the LRINEC score and other parameters combined with a low threshold for treatment should be maintained, especially in high-risk groups. Urgent referral to specialist surgical teams to ensure prompt diagnosis and treatment is essential to optimise outcomes in the face of this destructive infection.

## Data Availability

All data analysed in the current study are included in this article (presented in Tables 1 and 2).

## Abbreviations

PONF: Periorbital necrotizing fasciitis
BRVA: Best recorded visual acuity
LRINEC: Laboratory Risk Indicator for Necrotizing Fasciitis
PL: Perception of light
NPL: No perception of light
NF: Necrotizing fasciitis
IV: Intravenous
CRP: C reactive protein
WCC: White cell count
Hb: Haemoglobin
UK: United Kingdom
RAPD: Relative afferent pupillary defect
IOP: Intraocular pressure
PCR: Polymerase chain reaction
CT: Computerised tomography
HZO: Herpes zoster ophthalmicus
OCS: Orbital compartment syndrome
EK: Exposure Keratopathy
DM: Diabetes mellitus
GAS: Group A Streptococcus
SA: Staphylococcus aureus
NG: No growth
NA: Not available
SSG: Split-thickness skin graft
FTSG: Full Thickness Skin Graft
UO: Unable to Open
ENT: Ear Nose and Throat
